# Anæsthetics in Military Surgery

**Published:** 1862-06

**Authors:** P. W. Ellsworth

**Affiliations:** Late Brig. Surg. U. S. A.


ANÆSTHETICS IN MILITARY SURGERY.—*Messrs. Editors;* In your number for April fifth is an article headed "Fatal Anæsthesia," cautioning against the use of chloroform. It is a matter of the highest interest for the surgeon to know exactly what is the best agent for producing anæsthesia. I have been exceedingly cautious in the use of anæsthetics—in fact, perhaps too much so, for otherwise the first application of these agents in capital operations would probably have been by myself, having been on intimate terms with Dr. Horace Wells, the discoverer of their efficacy, and seeing his success, had been disposed to make more extended trial of them two years before the experiments in Boston. Since his discovery was established, the profession has been divided in opinion both as to the propriety of their use and the best agent. Having on the whole decided that they are useful, let us see which is the most advantageous. The result of the use of chloroform in the Crimean war was eminently satisfactory, apparently all that could be desired; but the experience of surgeons does not warrant the conclusions from thence deduced, as many cases have certainly proved fatal under the most guarded administration. Chloric ether has sometimes been substituted, but unless in combination with sulphuric ether or chloroform, it has not succeeded well in my hands, and has been laid aside. Sulphuric ether alone occasionally answered my purpose, but often failed; patients under its influence have not appeared to me as tranquil as when under chloroform, and sometimes it has produced no sensible effect when given to the extent of several ounces.

In this last class I have been obliged to resort to chloroform to accomplish my object. Nor can it be said that sulphuric ether is free from danger. In a patient of my own, where its use was not pushed to its utmost power, inability to converse, with partial paralysis of the tongue, followed, and continued for a week, showing a serious impression had been made upon the nervous system. In another instance, in the hands of a distinguished surgeon, its use was attended by fearful consequences, exciting in his mind great alarm for two days. Another argument against the use of sulphuric ether is the delay necessary in getting the patient under its influence, always to the surgeon the most tedious part of the operation. This is not, however, to be considered of weight, if the article is really so much superior in other respects to everything else, as is claimed by some, nor is the fact that it is very unpleasant, to be allowed much consideration, as these are of minor importance when life is at risk. I abandoned the use of simple chloroform many years ago, except where other anæsthetics failed, or where I was urged to use it by other surgeons or the patients themselves, having seen reported many fatal cases from its use, and having seen threatening symptoms in one of my own patients.

It would help us in arriving at a conclusion, which was on the whole the best of these two agents for general use, or the best in any particular case, if we knew exactly their respective modes of operation on the system. Do they each act in precisely the same manner? To me it appears that they do not; the sedative effect of chloroform on the heart is much greater than that of ether when given in moderate doses. Ether appears to act directly upon the blood, and indirectly upon the brain, the blood being very black when flowing from the dissevered arteries, and evidently poorly decarbonized; while with chloroform the impression would appear from its suddenness to be more direct upon the nervous system. Sulphuric ether, in moderate doses, while it tends to produce anæsthesia, excites the pulse; while chloroform producing anæsthesia seems to depress the vital power, sometimes to the point of fatal paralysis of the nerves supplying the heart and lungs.

If, now, anæsthesia can be produced with less exhaustion than results from the use of pure chloroform, or more speedily and effectually than from the use of simple sulphuric ether, it appears to me we should have attained one object, viz., safety and efficacy. Actuated by this reasoning, correct or not, I was early led, even before the combination was in use, so far as I know of, certainly before publicly known, to employ these articles in combination, the proportion being one of chloroform to two or three of sulphuric ether, according to the irritability of my patient. This proportion has been found abundantly successful, and I have relied upon it now for many years, having employed it in many severe and protracted operations; and I can not recall an unpleasant symptom, except in a very few instances of females, where a hysterical condition has lasted for an hour or two, and in one instance in a very irritable man, who was having his knee straightened after partial anchylosis; a somnolent condition persisted on several occasions in a light degree for several days. It would give me pleasure to get the views of practical surgeons upon this combination, one now chiefly used in the neighborhood where I practice. It does appear to me that the injurious effects of one article are counterbalanced by the other, while both are acting as anæsthetics, thus requiring much less of each than would be necessary if used alone. So far as my knowledge extends, embracing my own practice and that of others, the effect of the combination (used at once on mixing) has been all that could be wished for. In some instances, where persons were not susceptible, it has been necessary to push the inhalation even to the extent of stertor, yet in no such instance has the least unpleasant after-effect resulted. Used in this method, chloroform will deserve more praise than it has hitherto obtained, for alone it is certainly a dangerous article.

In closing, permit me to say that my experience coincides with that of Dr. Porter, U.S.A., who stated that in the Mexican war he found anæsthetics, particularly sulphuric ether, tend to produce hemorrhage. I have received a letter this day from the Pittsburg battle-field, from a surgeon who deplores the almost inevitable fatal result of amputations, as they are rapidly followed by secondary hemorrhage. In my own case often many more ligatures are necessarily applied than were requisite prior to the use of anæsthetics, although secondary hemorrhage has been rare. Anæsthetics must be used with more caution in an army hospital, since they necessarily poison the blood, already in a poor condition, and little able to react against the formidable injuries for the relief of which severe operations are requisite. In such I suspect chloroform will prove best. P. W. ELLSWORTH, M. D.,

Late Brig. Surg. U. S. A.

[*Medical and Surgical Reporter.*

